# Spectroscopic Ellipsometry Study of the Temperature Dependences of the Optical and Exciton Properties of MoS_2_ and WS_2_ Monolayers

**DOI:** 10.3390/ma17225455

**Published:** 2024-11-08

**Authors:** Hoang Tung Nguyen, Xuan Au Nguyen, Anh Tuan Hoang, Tae Jung Kim

**Affiliations:** 1 Institute of Materials Science, Vietnam Academy of Science and Technology, Hanoi 100000, Vietnam; 2 Department of Physics, Kyung Hee University, Seoul 02447, Republic of Korea; 3 School of Electrical and Electronic Engineering, Yonsei University, Seoul 03722, Republic of Korea

**Keywords:** MoS_2_ monolayer, WS_2_ monolayer, spectroscopic ellipsometry, temperature dependence, exciton behavior

## Abstract

The optical properties of MoS_2_ and WS_2_ monolayers are significantly influenced by fabrication methods, especially with respect to the behavior of excitons at the *K*−point of the Brillouin zone. Using spectroscopic ellipsometry, we obtain the complex dielectric functions of monolayers of these materials from cryogenic to room temperatures over the energy range 1.5 to 6.0 eV. The excitonic structure of each sample is analyzed meticulously by fitting the data to a standard analytical function to extract the energy positions of the excitons at each temperature. At low temperatures, excitonic structures are blue-shifted and sharpened due to the reduction in phonon noise and lattice distance. The excitons of monolayers fabricated by MOCVD separate into sub-structures at low temperatures, while monolayers grown by LPCVD and APCVD remain a single peak. The origin of these peaks as charged or neutral excitons follows from their temperature dependences.

## 1. Introduction

Transition metal dichalcogenides (TMDCs) have generated significant interest in recent years due to their extraordinary optical and electronic properties [1,2,3]. The fundamental features usually involve effects such as changing from indirect to direct bandgaps, enhancement of quantum yields, and modifications of spin-valley coupling by changing from multilayers to monolayers [4,5]. Among the 2D TMDCs, MoS_2_ and WS_2_ have been the most extensively investigated, including not only their fabrication methods [6,7] but also their structural [8], optical [9], and electronic properties [10,11]. Despite the enormous research conducted to understand the fundamental properties of excitons and trions (formed when an exciton binds to an additional electron or hole, creating a charged exciton) of these materials [12,13,14], the results remain diverse. The 2D nature of monolayer materials is significantly influenced by different fabrication methods and substrates [6,13,15], presenting difficulties in sample characterization. In fact, the energy separation between charged and neutral excitons is less than 50 meV. Hence, when several exciton peaks merge into one, the correct identification of the origin of these peaks can be ambiguous. 

Among the various optical methods used to characterize TMDC monolayers, spectroscopic ellipsometry (SE) is a powerful, non-destructive technique that allows for precise measurement of the dielectric functions (simultaneously obtaining the real and imaginary parts) of thin films [16,17,18,19]. When applied to monolayers, this technique can provide detailed insights into their optical properties across a wide energy range [10,20,21]. Although the excitonic behavior of various TMDCs has been thoroughly studied by photoluminescence (PL) and Raman spectroscopy under various conditions of temperature, strain [22], substrates [23], and growth methods [24,25], only a few studies have employed SE to characterize TMDCs [3,26,27,28,29,30,31]. Most of these works focus on extracting critical point energies from SE data of a single monolayer (MoS_2_ [26], WS_2_ [27], MoSe_2_ [30], WSe_2_ [29]) and identify the transitions on a calculated band structure. H.-S. Liu et al. measured the optical properties of TMDC monolayers by SE at various temperature and obtained the optical constants, temperature dependence of the bandgap, and thermo-optic coefficients [31]. X. Zhu et al. reported excitonic and critical point energies of MoS_2_ and WS_2_ monolayers obtained by SE at room temperature and the screening effect of their heterostructure [28]. However, a comprehensive investigation of exciton behavior in TMDC monolayers obtained by different fabrication approaches, especially under various temperature conditions, is not found. This knowledge is essential to understand band structure, excitonic effects and other quantum phenomena [32,33,34], and to design next-generation electronic and photonic devices that operate under various conditions, especially the harsh environment of space where devices are exposed to extreme heat and cold cycling. 

In this work, we demonstrate the use of SE to analyze the optical properties of MoS_2_ and WS_2_ monolayers prepared using different methods, specifically metalorganic chemical vapor deposition (MOCVD) for MoS_2_ and WS_2_, atmospheric chemical vapor deposition (APCVD) for MoS_2_, and low-pressure chemical vapor deposition (LPCVD) for WS_2_. The samples are measured at temperatures from cryogenic to 300 K in the energy range from 1.5 to 6.0 eV. Especially, the excitonic *A* and *B* structures, corresponding to the *K*-point transition in the Brillouin zone, are analyzed to identify and separate the structures at various temperatures. The MOCVD-grown monolayers exhibited clear formation and separation of exciton (*A*^0^, *B*^0^) and trion (*A*^−^, *B*^−^) peaks at low temperatures, helping to determine the trion-dominant behavior of the peaks. In contrast, the results of MoS_2_−APCVD and WS_2_−LPCVD showed only single *A* and *B* peaks even at temperatures lower than 100 K. In comparison to the results obtained from MOCVD samples, the excitons of MoS_2_−APCVD monolayers were identified as trion-dominant, while the excitons of WS_2_−LPCVD monolayers were identified as neutral exciton-dominant.

## 2. Materials and Methods

### 2.1. Sample Fabrication and Characterization

The large-area MoS_2_ and WS_2_ monolayer samples in this work were grown on Si substrates covered with approximately 300 nm of SiO_2_ via different approaches. The MoS_2_ monolayers were fabricated via MOCVD [35] and APCVD, respectively, denoted as M−MoS_2_ and A−MoS_2_. The WS_2_ monolayers were grown using MOCVD [27] and LPCVD, respectively, and are denoted as M−WS_2_ and L−WS_2_. We note that A−MoS_2_ and L−WS_2_ are grown by 2D Semiconductors Inc. and a study on M−WS_2_ has been presented in a previous work [27]. Some of these results are presented here for comparison and further discussion. To increase the reliability of the SE data analysis, all of the monolayers were transferred onto the sapphire substrate using polymethyl methacrylate [36], because backside reflection from the 300 nm SiO_2_ substrate otherwise causes crucial errors. We note that reducing the number of layers from three layers (monolayer/SiO_2_/Si) to only two layers (monolayer/sapphire) makes data analysis simpler and strengthens the validity of the results [26]. Microscope images of the samples on the sapphire substrate are presented in Figure S1. 

**Figure 1:**
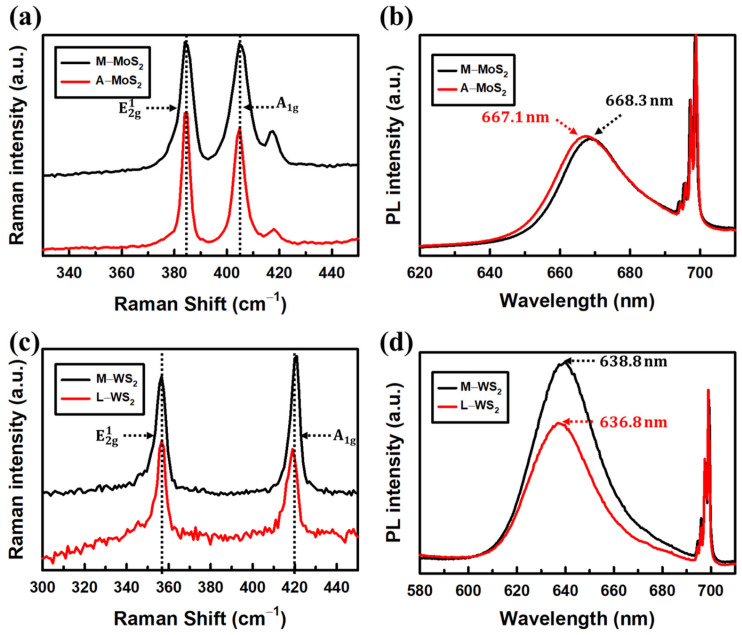
Typical Raman and PL spectra of MoS_2_ monolayer (**a**,**b**) and WS_2_ monolayer (**c**,**d**) samples performed with 532 nm laser excitation under ambient conditions. M-, L-, and A- stand for MOCVD, LPCVD, and APCVD fabrication methods, respectively.

After being transferred, the samples were characterized by Raman, PL, and SE at room temperature. Figure 1a,b present the Raman and PL spectra of the MoS_2_ monolayers, respectively. In Figure 1a, the $E_{2g}^{1}$–A_1g_ modes of M−MoS_2_ and A−MoS_2_ are found at 384.0–404.7 cm^−1^ and 383.9–404.9 cm^−1^, with the gap between two modes of 20.7 and 21.0 cm^−1^, respectively. These values are consistent with the MoS_2_ monolayer characteristics reported in Ref. [37]. In addition to the Raman spectrum, a signature PL peak of the MoS_2_ monolayer was also found at 668.3 and 667.1 nm for M−MoS_2_ and A−MoS_2_, respectively, confirming the high quality of the samples. Similarly, L−WS_2_ in this work shows signature $E_{2g}^{1}$–A_1g_ modes located at 356.6 and 419.0 cm^−1^, as shown in Figure 1c, along with a sharp PL peak at ~636.8 nm, as shown in Figure 1d. These values are consistent with M−WS_2_ monolayers presented in previous work [27]. A sharp PL structure of the monolayers next to the PL peaks at ~700 nm found in all the PL spectra emitted from the sapphire substrate indicate that the formed thin films are monolayers, since in the indirect bandgap properties of MoS_2_ and WS_2_ at bilayer and multilayer, the PL signals of the samples different from the monolayers are quickly quenched [38].

Next, a Woollam SE (model number: M2000-FI) with two focus lenses was used to measure various places with dimensions of 100 × 100 µm on the surface of the samples under atmospheric conditions to confirm the uniformity of the sample on a large scale, as shown in Figure S2 for M−WS_2_, with excellent matched data at different spots on the surface. The uniformity of other monolayers has been confirmed by the same approach and has had similar results. It was necessary to verify surface uniformity over large areas since focusing lenses cannot be used with the cryostat system.

### 2.2. SE Temperature Dependence

The experimental conditions for the temperature-dependence study are described in Ref. [27], where similar methods were used for 2D TMDC studies. Briefly, the monolayer samples were mounted on a cold-finger sample holder enclosed in a cryostat and cooled to below 70 K using liquid He. The base pressure was kept at 10^−8^ Torr to minimize artifacts resulting from condensation at low temperatures. Temperature monitoring was conducted using a silicon-diode thermometer on a dummy sample positioned at the corner of the cold finger. Incident light accessed the sample at an angle of 68.2° through stress-free fused-quartz windows. Dielectric function spectra were obtained from 1.5 to 6.0 eV at the lowest achievable temperatures to 300 K. A commercial dual-rotating-compensator ellipsometer (model RC2, J. A. Woollam Co., Inc. (Lincoln, NE, USA) at the Multi-Dimension Material Convergence Research Center of Kyung Hee University) was used for these measurements.

## 3. Results and Discussions

### 3.1. Analysis of <ɛ>

The pseudodielectric function <*ɛ*> spectra of the sample obtained by SE contain information of both the monolayers and the substrate. Therefore, a three-phase optical model including ambient/a TMDC monolayer/sapphire substrate was employed to extract the dielectric functions of the monolayers. Conventional approaches typically construct the dielectric functions of thin films using standard analytical functions such as Tauc–Lorentz, Lorentz, or Gaussian oscillators for each transition peak. However, in this work, the spectra measured at different temperatures are only slightly different from each other, making it difficult to trace using standard oscillators. Therefore, a higher-accuracy fitting method is required to preserve all the information of both real and imaginary parts of the dielectric functions for the second derivative analysis to be performed later in this study. Based on this issue, we employed a point-by-point fitting approach where the dielectric functions of the monolayers, including both the real and imaginary parts of *ɛ*, are obtained at each wavelength. In this approach, we fixed the thickness of MoS_2_ and WS_2_ monolayers at 0.7 and 0.8 nm, respectively [27,39,40]. Even if the surface roughness layer was not included in the optical model, the imaginary part of the dielectric function of all of the samples effectively resulted in a zero value below the bandgap region. This indicates the high quality and uniformity of the monolayers studied in this work. This also helps to reduce the number of free parameters required to describe the data.

**Figure 2:**
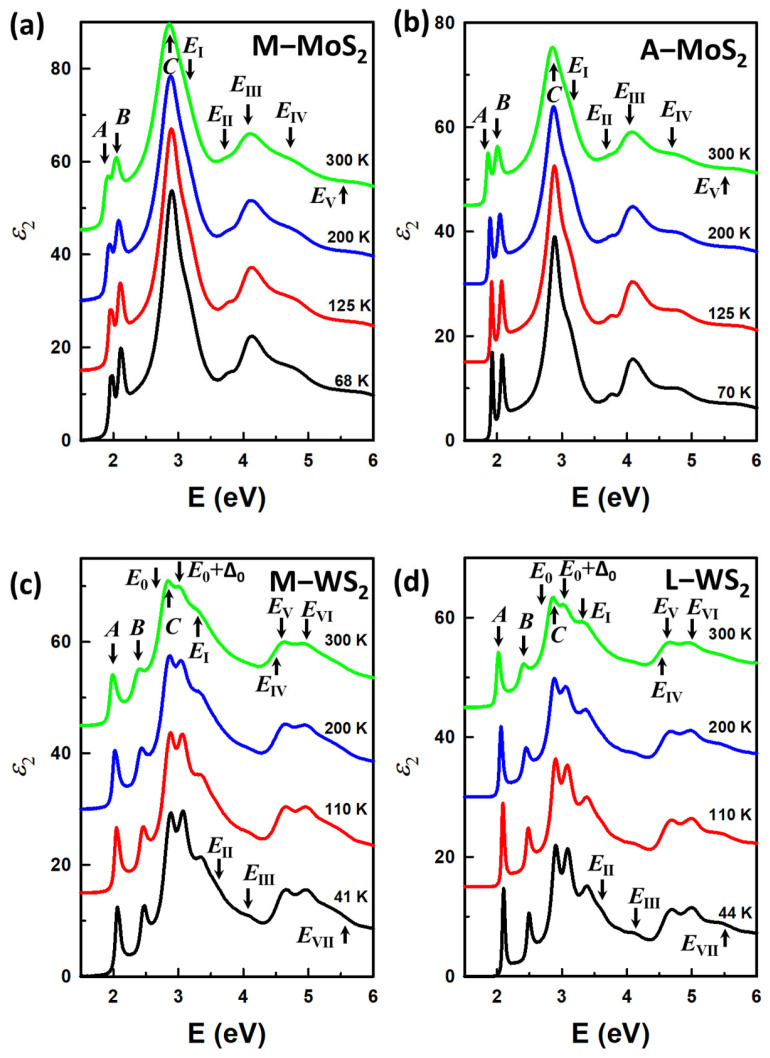
Imaginary parts of the dielectric function of (**a**) M−MoS_2_, (**b**) A−MoS_2_, (**c**) M−WS_2_, and (**d**) L−WS_2_ monolayers from 68 K, 70 K, 41 K, and 44 K, respectively, to 300 K. The spectra are offset by an increment of 15 for clarity.

Using this analysis approach, we extracted the dielectric functions of the M−MoS_2_, A−MoS_2_, M−WS_2_, and L−WS_2_ monolayers at each temperature, ranging from the lowest (68 K, 70 K, 41 K, and 44 K, respectively) to 300 K. The imaginary parts of the dielectric function of the mentioned monolayers are presented in Figure 2a–d for M−MoS_2_, A−MoS_2_, M−WS_2_, and L−WS_2_, respectively, at various temperatures. The spectra are offset by an increment of 15. At low temperatures, the critical point (CP) structures are blue-shifted and enhanced, with the appearance of new small CPs. These changes can be attributed to a significant reduction in electron–phonon interaction and the decrease in the lattice constant of TMDC materials at low temperatures, as figured out in Refs. [41,42]. Except for the *A* and *B* excitons, which will be discussed more thoroughly in this work, the data for the CPs (*C*, *E*_I_, *E*_II_, *E*_III_, *E*_IV_, and *E*_V_) in the MoS_2_ monolayers and CPs (*E*_0_, C, *E*_0_ + Δ_0_, *E*_I_ − *E*_VII_) in the WS_2_ monolayers, including their energies and identification on band structure, are in good agreement with previous work, and so are not discussed further [26,27]. A comparison of imaginary parts of the dielectric function of monolayers MoS_2_ and WS_2_ at low temperatures and at 300 K on full scale are presented in Figure S3. Besides, while CP structures become sharper with a reduction in temperature, CP structures are also observed to be sharper in the monolayers fabricated by APCVD (A−MoS_2_) and LPCVD (L−WS_2_) compared to their counterparts. This observation suggests better crystallization formed in the monolayers grown by the mentioned methods. It might come from the fact that APCVD/LPCVD samples usually have larger domain sizes (typically can be up to 50 µm) compared to those of MOCVD samples (typically up to 1 µm), as reported for various samples in Refs. [43,44]. 

**Figure 3:**
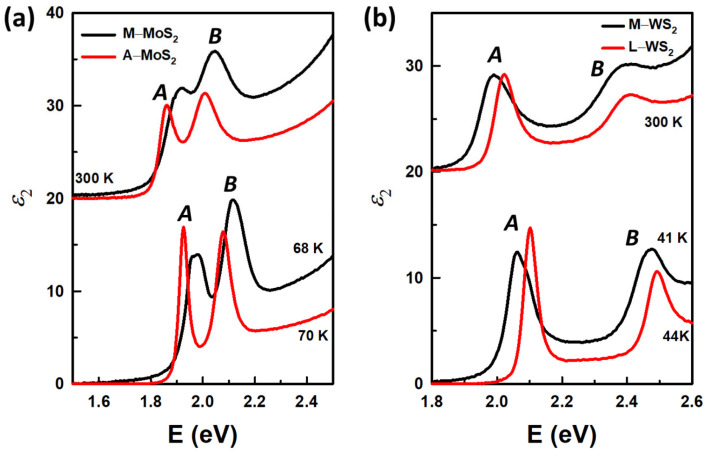
Imaginary parts of the dielectric function of monolayers (**a**) MoS_2_ and (**b**) WS_2_ at low temperatures and at 300 K on expanded scale around the regions of the *A* and *B* excitonic peaks.

In this work, we focused on the behaviors of the *A* and *B* excitons from each monolayer as a function of temperature and fabrication method. These excitons are well known to occur at energy ranges from 1.5 to 2.5 eV and from 1.8 to 2.6 eV for MoS_2_ and WS_2_ monolayers, respectively. For better comparison of the excitons at room and low temperatures, we replotted the *ɛ*_2_ spectra in Figure 3a,b for MoS_2_ monolayers and WS_2_ monolayers, respectively, with the upper spectra for 300 K and lower ones at the lowest temperatures. The structures in the *ɛ*_2_ spectra of all the monolayers shift to lower energy and decrease in amplitude as the temperature increases. We also observed the structures of M−MoS_2_ and M−WS_2_ show clear separation of the *A* and *B* excitonic peaks and are strongly asymmetric at low temperatures. In fact, M−WS_2_ *A* and *B* excitonic structures consist of negatively charged and neutral exciton peaks with the charged ones being dominant, as indicated in Ref. [27]. Meanwhile, A−MoS_2_ and L−WS_2_ show relatively sharp structures with no sign of peak separation. Measurements and analyses of the samples at low temperatures helped to reduce thermal effects, therefore allowing for more precise information on the excitonic states. This structure sharpening and separation cannot be observed in room temperature spectra, emphasizing the importance of measurements at cryostat temperatures. Even though reduced thermal noise allows for the separation of peaks at low temperatures, the exciton energies and structures cannot be qualitatively determined by simply inspecting the original spectra, as the exciton structures are notably asymmetric due to contributions from transitions across various regions of the Brillouin zone. Therefore, we employed a standard procedure to determine CP energies at different temperatures by analyzing the second derivatives of the *ɛ* spectra.

### 3.2. Exciton Energies

Exciton structures are usually hidden by overlapping and thermal noise. To resolve overlapped structures better even at low temperatures, second-derivative spectra, $\frac{d^{2}\varepsilon_{1}}{dE^{2}}$ and $\frac{d^{2}\varepsilon_{2}}{dE^{2}}$, were obtained using the Savitzky and Golay method for differentiation and appropriate smoothening [45]. The spectra were fit to a standard analytical CP expression [46]:

(1) $$\begin{array}{ccc} \frac{d^{2}\varepsilon }{d\omega^{2}} & = & n\left(n-1\right)A_{amp}e^{i\phi }{\left(\hbar \omega -E+i\Gamma \right)}^{n-2},\;\;\;n\neq 0,\\ & = & A_{amp}e^{i\phi }{\left(\hbar \omega -E+i\Gamma \right)}^{-2},\;\;\;\;\;\;\;\;\;n=0, \end{array}$$

where a CP is represented by the amplitude *A*_amp_, threshold energy *E*, broadening Γ, and phase *ϕ* as adjustable parameters. The exponent *n* has the values −1, −1/2, 0, and +1/2 for excitonic, 1, 2 (logarithmic), and 3D CPs, respectively. Both real and imaginary parts were fit simultaneously, and all the excitons were best fit with the *n* = −1 [26,27,47].

**Figure 4:**
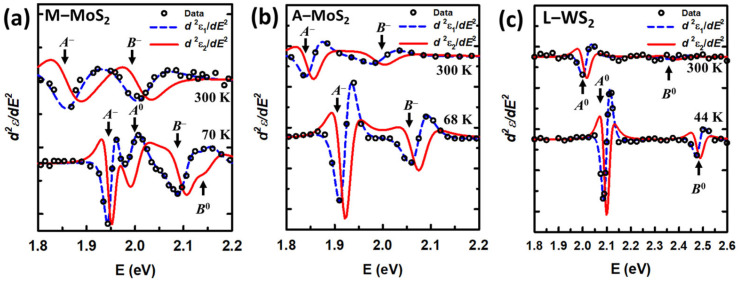
The best fit for $\frac{d^{2}\varepsilon_{1}}{dE^{2}}\;$ (dashed line) and $\frac{d^{2}\varepsilon_{2}}{dE^{2}}$ (solid line) from 1.8 to 2.2 eV for M−MoS_2_ (**a**) and A−MoS_2_ (**b**), and from 1.8 to 2.6 eV for L−WS_2_ (**c**). The data at each range is appropriately multiplied and the data for $\frac{d^{2}\varepsilon_{2}}{dE^{2}}$ (open circles) are not shown for clarity.

Figure 4a–c show the derivatives with their best fits for M−MoS_2_, A−MoS_2_, and L−WS_2_, respectively. The data of M−WS_2_ can be found in previous work [27]. Open circles represent the measured $\frac{d^{2}\varepsilon_{1}}{dE^{2}}$, while the dashed and the solid lines are the best fits to $\frac{d^{2}\varepsilon_{1}}{dE^{2}}\;$ and $\frac{d^{2}\varepsilon_{2}}{dE^{2}}$, respectively. The number of data points is reduced and the data for $\frac{d^{2}\varepsilon_{2}}{dE^{2}}$ are not shown for clarity. The spectra are offset by increments of 40,000. The blue-shift of CP energies and sharpening of the CP structures are clearly observed as the temperature decreases in all of the samples in Figure 4. The sharpening at low temperatures also helps to reveal the peak separation in M−MoS_2_ in the energy range of 1.8 to 2.2 eV.

The exciton energies of the monolayers determined at the lowest measured temperatures and at 300 K are listed in Table 1. In the room temperature MoS_2_ case, only two CPs at 1.86 and 2.01 are identified as *A* and *B* excitonic peaks. However, at 68 K, in addition to the blue-shift, these two CPs are separated into four CPs at 1.95, 2.00, 2.10, and 2.14 eV, being identified as *A*^−^ *A*^0^, *B*^−^, and *B*^0^, respectively. This identification agrees well with the binding energy of MoS_2_ (the energy required to separate an electron from its associated hole within an exciton), which is near 40 meV [12,48]. It is interesting to note that even with the second derivative analysis at low temperatures, no peak separation was found in A−MoS_2_ and L−WS_2_, in contrast to that of M−MoS_2_ in this work and M−WS_2_ in previous work [26]. The *A* and *B* exciton values of A−MoS_2_ are closely related to those of charged excitons in M−MoS_2_, while the *A* and *B* exciton energies of L−WS_2_ are as similar as those of M−WS_2_, leading to identification of these excitons as *A*^−^–*B*^−^ for A−MoS_2_ and *A*^0^–*B*^0^ for L−WS_2_. It means that for A−MoS_2_ and M−MoS_2_, trions can be found at all the temperatures, while for L−WS_2_, trions cannot be found even at the lowest temperature; for M−WS_2_, the *A^−^* trion can be found at all the temperatures and the *B*^−^ trion vanished at temperatures above 150 K. In a recent work by Zhu et al. [28], the authors measured MoS_2_ and WS_2_ monolayers by SE and reported transition energies of excitons *A*–*B* located at 1.88–2.00 eV for MoS_2_ and 2.02–2.40 eV for WS_2_. The origin of the excitons can hardly be identified due to the existence of only a single *A* and a single *B* peak. In comparison to the results obtained in this work (Table 1), *A* and *B* excitons of MoS_2_ monolayers might be recognized as trions, while *A* and *B* excitons of WS_2_ monolayers are neutral excitons. The temperature dependences of these excitons are presented in the next part, strengthening their identification of the origin. 

### 3.3. Temperature Dependence 

**Figure 5:**
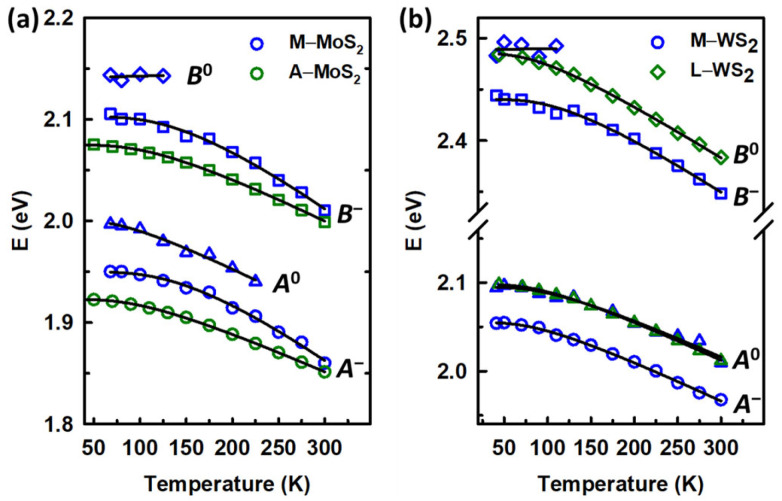
Temperature dependence of the CP energies (open symbols) and the best fit (solid lines) for excitons of (**a**) MoS_2_ monolayers and (**b**) WS_2_ monolayers. The data for M−WS_2_ are from Ref. [27].

By applying second-derivative analysis to the spectra at all of the temperatures, the exciton energy values are obtained, as shown in Figure 5a,b for the MoS_2_ and WS_2_ samples, respectively. The data for M−WS_2_ are from Ref. [27]. The temperature dependence of the exciton data of *A*^−^, *B*^−^, and *A*^0^ of A−MoS_2_, M−MoS_2_, and M−WS_2_, and *A*^0^ and *B*^0^ of L−WS_2_ are presented by fit results (solid lines) of the CP data to a phenomenological expression that contains the Bose–Einstein statistical factor for phonons [47,49]:

(2) $$E(T)\;=\;E_{B}-a_{B}\left[1+\frac{2}{e^{\Theta /T}-1}\right]$$

where Θ describes the mean frequency of the phonon and *a*_B_ the interaction strength between electrons and phonons. The mean phonon frequency indicates how large the contribution of the acoustic phonons is. In this model, the electron–phonon interactions are responsible for the shrinkage in the bandgap with an increase in temperature. The details of the theoretical model for the electronic band structure and phonon dispersion can be found in Ref. [31]. For the other exciton, which has negligible curvature (possibly due to the limited data), a linear equation [47,49] was applied:

(3) $$E\left(T\right)=E_{L}-\lambda T$$

where *E*_L_ is an adjustable parameter, while *λ* is the temperature coefficient *−dE/dT*. The best fit parameters are listed in Table 2.

In Figure 5a, the behaviors of the *A* and *B* excitons of MoS_2_ monolayers with temperature show a similar tendency, which certifies that their origins are related. The excitons of A−MoS_2_ follow the Bose–Einstein expression well, confirming the existence of single *A* and single *B* excitons even at low temperatures. By using second-derivative analysis and temperature-dependence fitting, we can confirm these excitons are *A*^−^ and *B*^−^ charged excitons, which originate from negatively charged trions consisting of two electrons and one hole. The positions of these excitons are slightly red-shifted at all temperatures relative to those of M−MoS_2_. However, the separation of these excitons (spin-orbit splitting at maximum valence band at *K* valley) of A−MoS_2_ remains at 150 meV, which is the same value as that of M−MoS_2_ and agrees well with previous reports [50,51]. The fact that the fabricated A−MoS_2_ shows strongly trion-dominant behavior with no existence of neutral exciton peaks, while M−MoS_2_ shows trion-dominant with a separation of neutral-charged exciton peaks at low temperatures (below 250 K for *A*^0^ and below 150 K for *B*^0^), might relate to an influence of defect densities and impurities of the samples and/or differences in the local built-in strain originating from different growth methods. 

It should be noted that, without careful experiments and analysis by temperature dependence, it is impossible to determine the origin of the excitonic peaks of A−MoS_2_ and M−MoS_2_, since each sample contains only single *A* and *B* peaks at relatively close energies in room-temperature measurements. The energy difference between *A*^0^ and *A*^−^ (trion binding energy) appears to be temperature-independent and remains constant at ~45 meV for MoS_2_. This value is in good agreement with the calculation of 43 meV by Matthias Drüppel et al. [13]. In MoS_2_, negative trions are commonly observed due to high electron density, which is often doped unintentionally, originating from sulfur vacancies, and too substantial to be fully neutralized by electronic back-gating of substrates. These excess electrons facilitate the formation of trions by binding with excitons, resulting in a significant number of trions even at room temperature [52]. 

The temperature dependences of the excitonic peaks of L−WS_2_ are presented in Figure 5b, in comparison to that of M−WS_2_ from the previous work [27]. In contrast to the excitonic behavior of A−MoS_2_ and M−MoS_2_ in this work, and M−WS_2_ in the previous work, which are all trion-dominant, L−WS_2_ shows single excitonic peak for each *A* and *B* exciton at the energies coinciding with those of *A*^0^ and *B*^0^ of M−WS_2_. In the previous work for M−WS_2_, due to peak separation, one can easily recognize the origin of each of the four excitons *A*^−^, *A*^0^, *B*^−^, and *B*^0^. With increasing temperature, the *A*^−^ and *B*^−^ excitons remain dominant, while the *A*^0^ structure decreases rapidly and *B*^0^ quickly vanished above ~150 K. They are thus hardly recognizable without a temperature study. 

*A* and *B* excitons for L−WS_2_ in this work show total dominance of neutral exciton peaks without recognizable separation of trion structures even at cryogenic temperatures. The spin-orbit splitting of L−WS_2_ in this work remains at 382 meV, similar to that reported in Ref. [27]. The dominance of the neutral excitons of L−WS_2_ in this work, as opposed to the trion dominance of M−WS_2_ reported in the previous work, may come from differences in fabrication approaches leading to significantly sharper exciton peaks and fewer structural defects, which are a well-known source of trion formations, as depicted previously [14,53,54]. It is noteworthy that the *B*^0^ exciton in the previous work could not be properly fitted due to lack of data after 120 K. This has now been addressed in this work. Last but not least, the knowledge of the thermo-optic properties of TMDC monolayers acquired in this work would be helpful in designing all-optical switch devices [55,56].

## 4. Conclusions

We present a comparison study of the optical properties of MoS_2_ monolayers grown using MOCVD (M−MoS_2_) and APCVD (A−MoS_2_), and WS_2_ monolayers fabricated using MOCVD (M−WS_2_) and LPCVD (L−WS_2_). The monolayers were measured by SE at temperatures from cryogenic to 300 K in an energy range from 1.5 to 6.0 eV. The excitonic energies at each temperature were studied by fitting the second derivatives of the obtained spectra to a standard analytical CP expression to identify and separate the structures at various temperatures. While the MOCVD monolayers clearly show the formation and separation of exciton (*A*^0^ and *B*^0^) and trion (*A*^−^ and *B^−^*) peaks with trion-dominant behavior, in A−MoS_2_ and L−WS_2_ we found only single *A* and *B* peaks. The A−MoS_2_ monolayer was identified as trion-dominant and the L−WS_2_ monolayer was identified as neutral exciton-dominant as a result of the data presented here. 

## Figures and Tables

**Table 1:** Exciton energies at the lowest measured temperature and at 300 K of the monolayers.

Exciton Energies	A−MoS_2_		M−MoS_2_		L−WS_2_		M−WS_2_^1^	
	70 K	300 K	68 K	300 K	44 K	300 K	41 K	300 K
*A* ^−^	1.92 ± 0.01	1.85 ± 0.03	1.95 ± 0.01	1.86 ± 0.03	--	--	2.05 ± 0.01	1.96 ± 0.02
*A* ^0^	--	--	2.00 ± 0.02	--	2.10 ± 0.02	2.01 ± 0.02	2.09 ± 0.01	2.00 ± 0.02
*B* ^−^	2.08 ± 0.02	2.00 ± 0.04	2.10 ± 0.01	2.01 ± 0.02	--	--	2.44 ± 0.01	2.34 ± 0.02
*B* ^0^	--	--	2.14 ± 0.02	--	2.48 ± 0.01	2.38 ± 0.04	2.48 ± 0.02	--

^1^ Data from Ref. [27].

**Table 2:** The best-fitting parameters of the temperature dependences of the CPs of the monolayers.

Sample	Exciton	*E*_B_ (eV)	*a*_B_ (meV)	Θ (K)	*E*_L_ (eV)	*λ* (10^−4^ eVK^−1^)
A−MoS_2_	*A* ^−^	1.99 ± 0.01	67 ± 3	318 ± 8	--	--
	*B* ^−^	2.16 ± 0.01	87 ± 5	361 ± 12	--	--
M−MoS_2_	*A* ^−^	2.14 ± 0.03	191 ± 33	505 ± 42	--	--
	*A* ^0^	2.06 ± 0.03	60 ± 34	249 ± 93	--	--
	*B* ^−^	2.28 ± 0.03	186 ± 36	488 ± 47	--	--
	*B* ^0^	--	--	--	2.14 ± 0.01	0.26 ± 0.01
L−WS_2_	*A* ^0^	2.17 ± 0.01	69 ± 3	287 ± 9	--	--
	*B* ^0^	2.56 ± 0.03	73 ± 4	267 ± 9	--	--
M−WS_2_^1^	*A* ^−^	2.11 ± 0.01	63 ± 6	266 ± 17	--	--
	*A* ^0^	2.16 ± 0.02	73 ± 22	314 ± 59	--	--
	*B* ^−^	2.55 ± 0.02	112 ± 23	374 ± 42	--	--
	*B* ^0^	--	--	--	2.49 ± 0.01	0.10 ± 0.01

^1^ Data from Ref. [27].

## Data Availability

The original contributions presented in the study are included in the article/Supplementary Material, further inquiries can be directed to the corresponding authors.

## Supplementary Materials

The following supporting information can be downloaded at: https://www.mdpi.com/article/10.3390/ma17225455/s1, Figure S1: Optical images of the monolayers (a) M−MoS_2_, (b) A−MoS_2_, and (c) L−WS_2_ on sapphire substrate.; Figure S2: Measured Ψ and Δ of monolayer M−WS_2_ at various spots by focused beam SE.; Figure S3: Imaginary parts of dielectric function of monolayers (a) MoS_2_ and (b) WS_2_ at low temperature and at 300 K.
