# Supplementary material for: Spectroscopic Ellipsometry Study of the Temperature Dependences of the Optical and Exciton Properties of MoS2 and WS2 Monolayers
Source: Materials (Basel). 2024 Nov 8;17(22):5455. doi: 10.3390/ma17225455 (PMC11595284; doi:10.3390/ma17225455)
Supplement: Supplementary file 1 [file materials-17-05455-s001.zip › materials-3261916-supplementary.pdf]

Supplementary materials

Spectroscopic Ellipsometry study of the Temperature Dependences of the Optical and Exciton Properties of MoS_2 and WS_2 monolayers

Hoang Tung Nguyen[1*], Xuan Au Nguyen[2], Anh Tuan Hoang[3], and Tae Jung Kim[2*]

[1] Institute of Materials Science, Vietnam Academy of Science and Technology, Hanoi, Vietnam
[2] Department of Physics, Kyung Hee University, Seoul, Republic of Korea
[3] School of Electrical and Electronic Engineering, Yonsei University, Seoul, Republic of Korea
* Correspondence: tungnh@ims.vast.ac.vn; tjkim@khu.ac.kr;

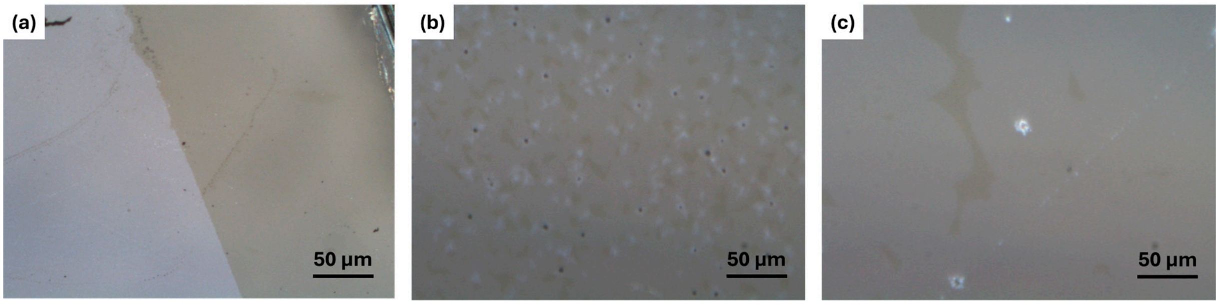

Figure S1. Optical images of the monolayers (a) M-MoS_2, (b) A-MoS_2, and (c) L-WS_2 on sapphire substrate.

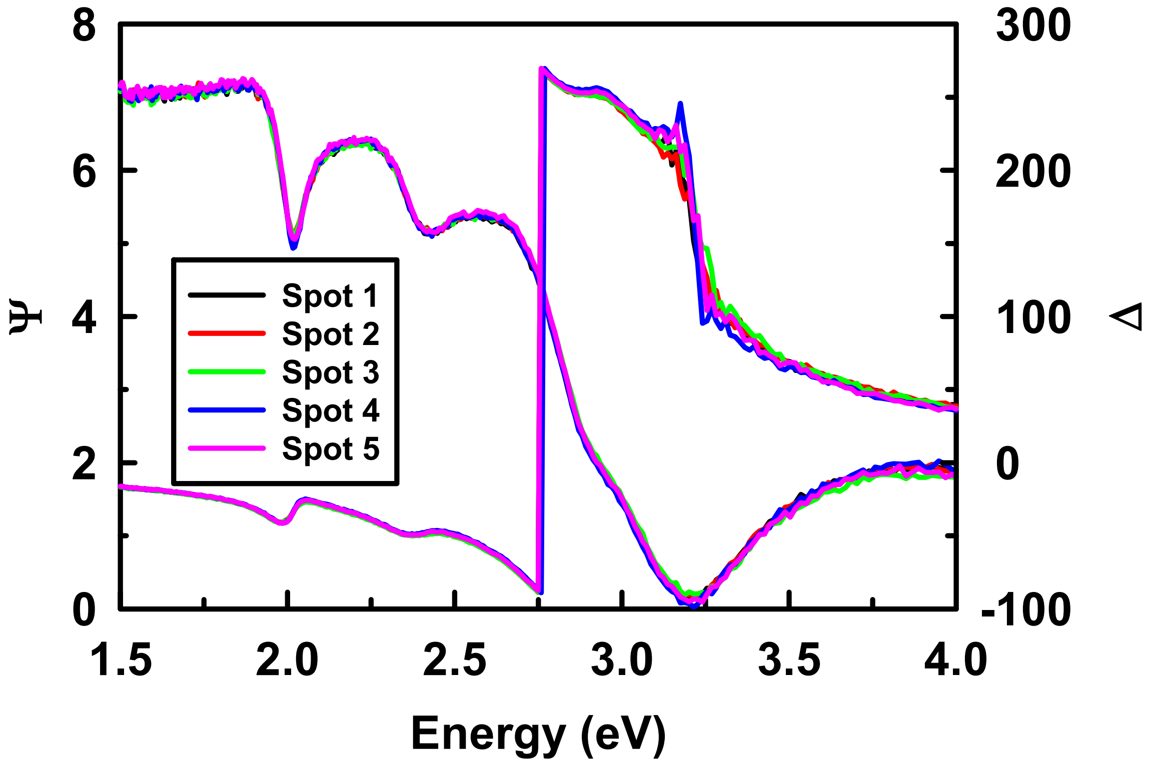

Figure S2. Measured Ψ and Δ of monolayer M-WS_2 at various spots by focused beam SE.

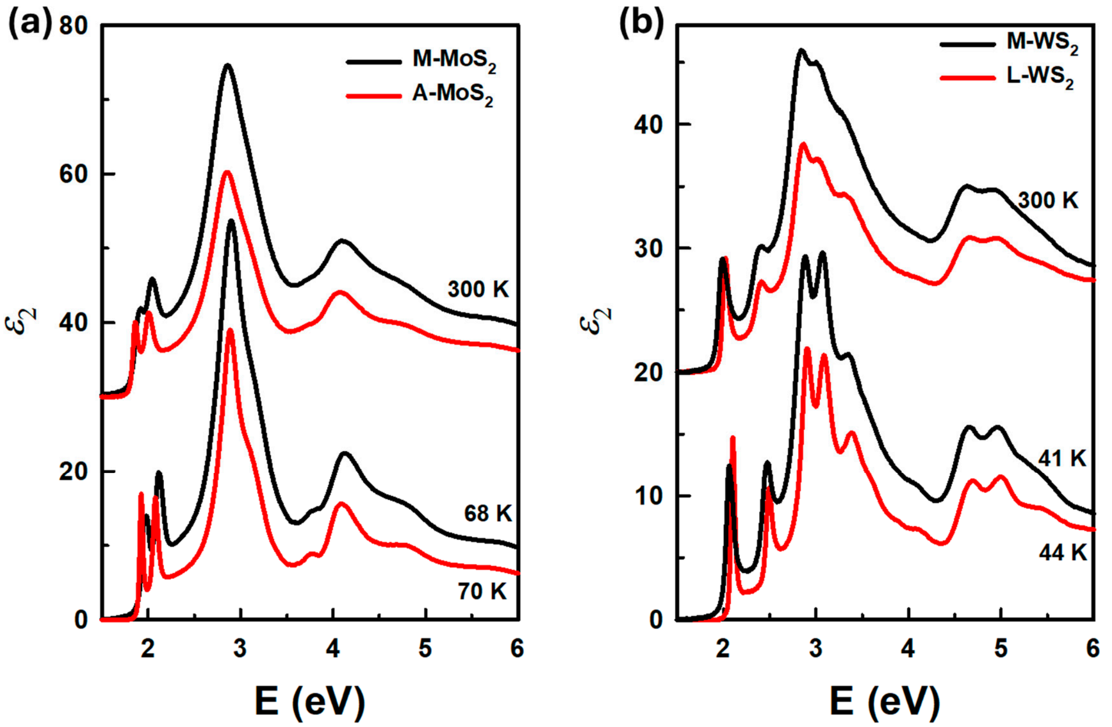

Figure S3. Imaginary parts of dielectric function of monolayers (a) MoS_2 and (b) WS_2 at low temperature and at 300 K.
